# Unifocal and multifocal chronic non-bacterial osteomyelitis (CNO) in children

**DOI:** 10.1186/1546-0096-9-S1-P32

**Published:** 2011-09-14

**Authors:** G Zanon, A Insalaco, G Martini, S Corradin, P Zucchetta, L Tanturri, M Garganese, F De Benedetti, F Zulian

**Affiliations:** 1 Dept. of Pediatrics, University of Padua, Italy; 2 IRCCS Ospedale Pediatrico Bambino Gesù, Rome, Italy; 3 Dept. of Radiology, University of Padua, Italy; 4 Dept. of Nuclear Medicine, University of Padua, Italy

## Background

Chronic non bacterial osteomyelitis (CNO) is a rare condition in children and little is known about its clinical assessment and course.

## Aim

To describe the clinical characteristics and long-term outcome of pediatric patients with CNO.

## Methods

We retrospectively evaluated patients with sterile bone inflammation, lasting longer than 6 month, referred to two tertiary care pediatric rheumatology units. Information on family history, clinical features at disease onset and course, laboratory and outcome were collected. A comparison between unifocal (UF-CNO) and multifocal course (MF-CNO), based on bone scintiscan result, was performed.

## Results

29 CNO patients entered the study. Ten had UF-CNO, 19 had MF-CNO, mean age at disease onset 9,2 years (range 0,8-17), males (52%). Disease duration at diagnosis was longer in pts with UF-CNO (11,1 vs 4,6 months). Localized bone pain was the leading symptom at onset in all patients; systemic symptoms, such as fever and fatigue, were more frequent in MF-CNO. 30% presented associated skin disease and positive family history for autoimmune disease. At onset WBC normal, CRP was elevated in 38%, ESR in 72%, especially in MF-CNO. Scintiscan allowed us to identify multiple lesions in five patients with one-site symptoms. MF-CNO involve more frequently the lower limbs than the UF-CNO (18/19 vs 4/10, p 0,001). After three years follow up, 70% of patients had no symptoms and 90% were off-therapy. UF-CNO developed complications, such as hyperostosis, vertebral collapse or limb dysmetry, significantly more often than MF-CNO (p 0,002).

## Conclusion

UF-CNO and MF-CNO belong to the same spectrum but present different clinical features and outcome.

